# Phenotypic Analysis of a Population of IgA^+^ Cells in the Follicle-Associated Epithelium of Mouse Peyer's Patches

**DOI:** 10.1371/journal.pone.0124111

**Published:** 2015-04-20

**Authors:** Maria Olga Hernandez, Nicholas J. Mantis

**Affiliations:** 1 Division of Infectious Diseases, Wadsworth Center, New York State Department of Health, Albany, New York, 12208, United States of America; 2 Department of Biomedical Sciences, University at Albany, Albany, New York, 12208, United States of America; Indian Institute of Science, INDIA

## Abstract

The follicle-associated epithelium (FAE) selectively transports prions, viruses, pathogenic bacteria, commensal microflora, and even secretory IgA (SIgA)-immune complexes from the intestinal lumen to underlying gut-associated lymphoid tissues like Peyer’s patches. The FAE consists of a single layer of columnar epithelial cells that includes enterocytes and M (microfold) cells, intermingled with dendritic cells (DCs), macrophages, and naïve and memory B and T lymphocytes. In this report we describe a population of IgA^+^ cells that reside within and immediately below the FAE in mouse Peyer’s patches. Immunofluorescence microscopy analysis indicated that the FAE-associated IgA^+^ cells were negative for surface antigen markers specific for B cells (B220), T cells (CD3), DCs (CD11c), and plasma cells (CD138). The IgA^+^ cells were also negative Ki-67 and IRF4, indicating that they are not mature B cells or plasma cells. The IgA^+^ cells were, however, often found in close proximity to DCs, leading us to speculate that the population of IgA^+ ^ cells in the FAE constitutes an atypical subset of B cells involved in mucosal antigen surveillance and/or immune recall.

## Introduction

Mucosal antibody responses associated with protective immunity to enteric diseases like cholerae, dysentery and rotavirus originate within gut-associated lymphoid tissues (GALT) known as Peyer’s patches. Peyer’s patches, which by definition consist of aggregates of five or more lymphoid follicles, are situated along the length of the small intestine, with increased numbers in the ileum [1, 2]. Peyer’s patches are unique among secondary lymphoid tissues in that they lack afferent lymphatics. Consequently, antigens in the Peyer’s patch are not derived from interstitial fluids; rather, they are sampled directly from the intestinal lumen by the so-called follicle-associated epithelium (FAE). The FAE is columnar in nature and consists of two primary epithelial cell types involved in antigen sampling: enterocytes and microfold (M) cells. M cells are highly specialized for the uptake of particulate antigens, including viruses, bacteria and even yeast-sized particles [3–6]. M cell apical membranes are highly endocytic and devoid of microvilli, while their basolateral membranes are invaginated to form a pocket that is populated by B and T lymphocytes (mainly memory cells), as well as dendritic cells (DCs) and macrophages [7–11]. FAE enterocytes also play a role in the uptake and transepithelial transport of certain antigens such as prions, although the specific mechanisms involved in these processes are not well understood [12, 13].

Following trans-FAE transport, antigens are captured by an underlying network of DCs (and occasional macrophages), which facilitate antigen presentation and the onset of antigen-specific T and B cell responses, as well as oral tolerance [8, 14–19]. The microenvironment of Peyer’s patches is such that B cells preferentially class switch to IgA (rather than IgG) and express joining or “J” chain. B cells, including plasma cell precursors, exit Peyer’s patches via the efferent lymphatics, drain to the mesenteric lymph nodes and ultimately home to the surrounding intestinal lamina propria where they differentiate into IgA secreting plasma cells [2, 20, 21]. Locally produced dimeric and polymeric IgA is then transported across the epithelium and into intestinal secretions by the polymeric immunoglobulin receptor (pIgR) [22]. Once in the intestinal lumen, SIgA functions in preventing enteric pathogens as well as certain commensal from colonizing or invading the intestinal epithelium [23–25].

Despite the FAE’s central role in initiating mucosal antibody responses, the exact subsets of lymphocytes that reside within this unique epithelial interface have not been fully characterized. While it is known that FAE-associated T cells are mostly CD4^+^ (and not CD8^+^) and, in humans, the majority display CD45RO, a surface marker typical of memory T cells [7, 26]. The B cell population in the FAE is a mixture of naïve (SIgD+) and memory (SIgD-) cells that are proposed to originate from the underlying B cell follicles [27]. Based on co-stimulatory molecule expression and in vitro co-culture assays, Brandtzaeg and colleagues have proposed that M cells pockets are in fact extensions of germinal centers [2, 10, 11, 28] and that memory B cells within this niche are actively engaged in sampling luminal antigens and presenting them to adjacent T cells, which in turn could promote memory B cell survival and proliferation [10, 11].

In a recent study, we noted a population of IgA^+^ cells situated within the FAE, possibly in association with M cells, of mouse Peyer’s patches (M.O. Hernandez, S. Ahlawat, M. De Jesus and N. Mantis, unpublished results; [29]. While Farstad and colleagues have reported having observed IgA^+^ cells associated with M cells in human Peyer’s patches, no attempts were made to determine their relative frequency in the FAE, to establish their surface antigen profile, or examine whether the cells are responsive to external stimuli [7]. It is known that specific Toll-like receptor agonists, as well as the mucosal adjuvant cholera toxin (CT) influence the migration of DCs [14, 30, 31] as well as B cells [1] in/out of the FAE and SED. To begin to address these questions, we performed multicolor immunofluorescence confocal microscopy of mouse Peyer’s patch cryosections using a panel of antibodies directed against clusters of differentiation (CD) antigens specific for a variety of cell subsets known to be associated with cells in the GALT. We also examined the frequency of IgA^+^ cells in polymeric immunoglobulin receptor (pIgR) knock-out mice (which lack secretory IgA). Collectively our data demonstrate that IgA^+^ cells are intimately associated with M cells and DCs and are likely an atypical subset of lymphocytes cells involved in mucosal antigen surveillance and/or immune recall.

## Materials and Methods

### Chemicals and reagents

Cholera toxin (CT) from *Vibrio cholerae* type Inaba 569B was obtained from Calbiochem (Billerica, MA). Fluorophore-labeled primary and secondary antibodies were purchased from BD Biosciences (Franklin Lakes, NJ), eBioscience (San Jose, CA) and other manufactures, as indicated in Table 1.

**Table 1:** Antibodies used for immunostaining in this study.

Marker (fluorophore)	Cell Type	Vendor
CD45R/B220—(APC)	B cells	BD Biosciences
CD3—(FITC)	T cells	BD Biosciences
IgA—(FITC)	IgA	BD Biosciences
IgA—(TRITC)	IgA	Southern Biotech
IgG—(DayLight 550)	IgG	Abcam
IgM-(FITC)	IgM	BD Biosciences
E-Cadherin—(FITC)	Epithelial cell junctions	BD Biosciences
CD 196—(Alexa Fluor 647)	CCR6	BD Biosciences
CD199—(APC)	CCR9	eBioscience
CD11c—(PE)	DCs	eBioscience
CD11b—(APC)	Monocytes/Macrophages	BD Biosciences
CD19—(APC)	B cells	BD Biosciences
CD138—(APC)	Plasma Cells	BD Biosciences
IRF4—(PE)	Mature Plasma Cells	eBioscience
CCL20/MIP3α—(AlexaFluor 555)	Chemokine	Bioss
Ki67—(FITC)	Plasmablasts	Abcam

### Mouse gavage and tissue collection

BALB/c female mice (8–12 weeks old) were purchased from Taconic (Hudson, NY). C57BL/6Tc female mice were purchased from University of Missouri (Columbia, MO). The pIgR^-/-^ mice [B6.129P2-*Pigr* ^*tm1Fejo*^/Mmmh], which are devoid of the polymeric immunoglobulin receptor (pIgR) and therefore unable to transport polymeric IgA or IgM into intestinal secretions, were purchased from Jackson Labs (Bar Harbor, ME). Mice were housed under conventional, specific pathogen-free conditions and were treated in compliance with the Wadsworth Center’s Institutional animal Care and Use Committee (IACUC) guidelines. Mice were euthanized by carbon dioxide asphyxiation and PP were harvested for immunostaining as described [4, 29]. Briefly, Peyer’s patches were snap-frozen in liquid nitrogen and embedded in TissueTek1 Optimal Cutting Temperature (OCT) Compound (Sakura Finetek, Torrance, CA, USA). Cryosections (20μm) were prepared with a Leica CM3050S cryomicrotome (Leica, Wetzlar, Germany) and were air dried overnight at 4^°^C before immunostaining.

### Ethics Statement

Experiments described in this study that involve mice were reviewed and approved by the Wadsworth Center’s Institutional Animal Care and Use Committee (IACUC) under protocol #12–428. Mice were euthanized by carbon dioxide asphyxiation followed by cervical dislocation, as recommended by the Office of Laboratory Animal Welfare (OLAW), National Institutes of Health. The Wadsworth Center complies with the Public Health Service Policy on Humane Care and Use of Laboratory Animals and was issues assurance number A3183-01. Moreover, the Wadsworth Center is fully accredited by the Association for Assessment and Accreditation of Laboratory Animal Care (AAALAC). Obtaining this voluntary accreditation status reflects that Wadsworth Center’s Animal Care and Use Program meets all of the standards required by law, and goes beyond the standards as it strives to achieve excellence in animal care and use.

### Immunofluorescence microscopy of Peyer’s patch tissue

Peyer’s patch cryosections were stained as described [4, 29]. Sections were fixed for 2 min in acetone, washed in PBS-Tween 20 (PBS-T; 0.05%, vol/vol), encircled with an ImmEdge hydrophobic pen (Vector Labs, Burlingame, CA) and then blocked with 2% goat serum in PBS for 30 min at 37^°^C. Slides were rinsed for 10 sec in PBS containing 0.5% Tween (PBS-T) and Fc receptors were then blocked with supernatants from the ATCC 2.4.G2 cell line that blocks FcγRII for 10 min at 37^°^C. Sections were stained for 1 hour at 37^°^C in a humidified chamber with an antibody cocktail of the selected antibodies to study. Sections were fixed with 4% paraformaldehyde for 4 min at RT, washed once with PBS-T for 1 min. Slides were mounted with ProLong Gold Antifade reagent (Invitrogen, Grand Island, NY).

### Image acquisition and analysis

Confocal images were collected on a Leica SP5 ABOS confocal system using a 10X objective lens (NA 0.4) and a 2X digital zoom (Leica, Wetzlar, Germany). Data sets were collected as a series of optical sections using 0.4 μm steps. The voxel size was 757×757×1300 nm. Channels were collected sequentially, to minimize possible spectral overlap. Color digital photomicrographs were generated using an Olympus (IX 70) microscope (Hawthorn, NY) equipped with 10X and 20X (0.3,0.45 NA) objectives lenses. Images were captured with a Retiga 2000R camera equipped with a RGB filter. Q Capture Pro imaging software (Q-Imaging, Surrey, BC Canada) was used to control the camera, filter wheel and shutter. Brightness and normalization was accomplished using Fiji software.

### Statistical analysis

The Student’s t test was used to determine statistical significance between the numbers of IgA+ cells in the FAE of WT and pIgR-/- mice (GraphPad Prism, v5, San Diego, CA).

## Results and Discussion

### Identification of an IgA^+^ cells in the FAE of mouse Peyer’s patches

**Fig 1:**
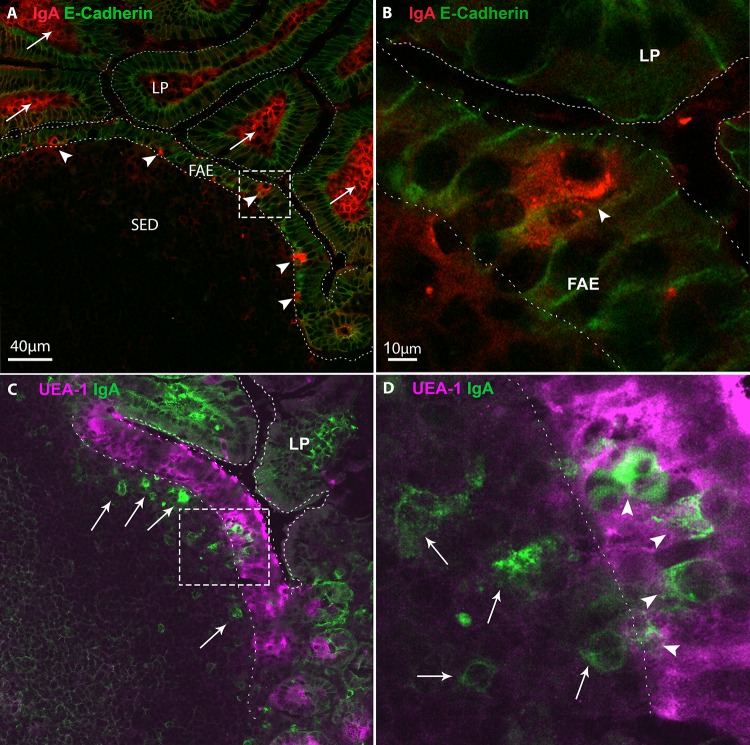
IgA^+^ cells in the FAE of mouse Peyer’s patches. Cryosections of BALB/c Peyer’s patches were stained with antibodies directed against IgA (red, panels A, B; green, panels C, D), E-cadherin (green, Panel A, B) or the lectin UEA-1 (magenta, panels C,D), and then visualized by CLSM. (**A, B**) IgA+ cells are distributed within LP (arrows) and in FAE (arrowheads). The dashed box in Panel A is magnified in panel B. **(C, D)** In the FAE, IgA + cells are associated with UEA-1 positive cells, indicting that they are likely M cells. The dashed box in Panel C is magnified in panel D. Abbreviations: SED, subepithelial dome; FAE, follicle-associated epithelium.

**Fig 2:**
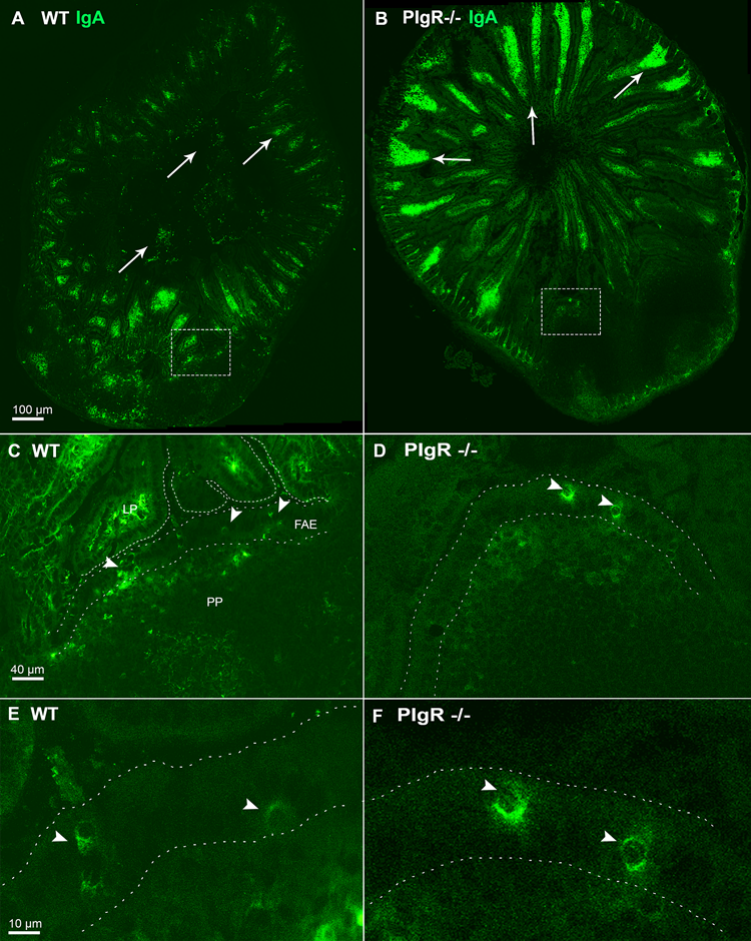
IgA^+^ cells are present in the FAE in the Peyer’s patches of pIgR knock-out mice. Cryosections of C57BL/6 (panels A, C, E) or pIgR^-/-^ (panels B, D, F) mouse intestinal tissues containing Peyer’s patches were stained with antibodies directed against IgA (green) and then visualized by CLSM. Panels C and D are magnifications of the dashed boxes in Panels A and B, while Panels E and F are magnifications of Panels C and D. IgA+ cells are distributed within LP (arrows) and in the FAE (arrowheads). In panels C-F the FAE is delineated with dashed lines. Scale bars: panel A and B, 100 μm; panel C and D, 40 μm; panel E and F, 10 μm.

We stained cryosections of naïve BALB/c mice Peyer’s patches with polyclonal antibodies against IgA, and made use of an antibody against E-cadherin to delineate the lateral aspects of villus enterocytes and FAE enterocytes. As expected, IgA^+^ staining was observed in the LP, the primary site where IgA plasma cells reside (Fig 1A–1D)[2]. In the villi, IgA^+^ plasma cells were restricted to lamina propria, and were not observed in the epithelium. Examination of lymphoid follicles revealed that IgA+ staining was absent in the germinal centers (GC) and the follicle but occasionally observed in the interfollicular regions (IFR) (Figs 1A; 2A, 2C and 2E). However, notable IgA staining was routinely observed in the SED and throughout the FAE from the crypts to the dome (Fig 1A–1D). Staining tissues with UEA-1, a known marker of M cells in BALB/c mice, indicated that the IgA^+^ cells were likely situated within or adjacent to M cell basolateral pockets (Fig 1, arrowheads). IgA^+^ cells were often observed situated in close proximity to the basal lamina (Fig 1, asterisk), suggesting possible migration of cells into or out of the FAE. IgA^+^ cells were present in the FAE of Peyer’s patches sampled from the entire length of the small intestine (data not shown), although we primarily focused our attention on the 2–3 most proximal Peyer’s patches for the sake of this study. On average there were 4–5 IgA^+^ cells in the FAE in a single 20 μm thick cryosection and slightly more IgA+ cells present in the SED than in the FAE. Additional immunostaining revealed that IgM^+^ cells were also present in the SED and FAE but only rarely in the lamina propria (S1 Fig). IgG^+^ cells were prevalent in the lamina propria and the SED, but rarely (if ever) observed in the FAE (S2 Fig).

It was possible that IgA+ cells in the FAE and SED were the result of cells simply having “captured” SIgA following trans-epithelial transport of antibody from the intestinal lumen by M cells, as we and other have reported [32–34]. To test this hypothesis, we performed immunostaining on Peyer’s patches tissues from pIgR knock-out (pIgR^-/-^) mice, which are devoid of SIgA due to a mutation the polymeric IgA receptor [35]. As compared to WT mice, the pIgR^-/-^ mice had increased IgA fluorescence labeling within the lamina propria, which is attributable to the local accumulation of IgA due to the absence of pIgR-mediated transport (Fig 2). Examination of the Peyer’s patches of the pIgR^-/-^ mice identified IgA^+^ cells in the FAE, indicating that the IgA-positive staining observed in this microcompartment is not due to SIgA being retro-transported from the lumen. It is interesting to note, however, that the number of IgA^+^ cells found at the FAE of pIgR^-/-^ mice was slightly reduced (3–4 per FAE) compared to the total number of IgA^+^ cells found at the FAE of Peyer’s patches in wild type BALB/c and C57BL/6 mice.

### Phenotypic analysis of IgA^+^ cells in the FAE

**Fig 3:**
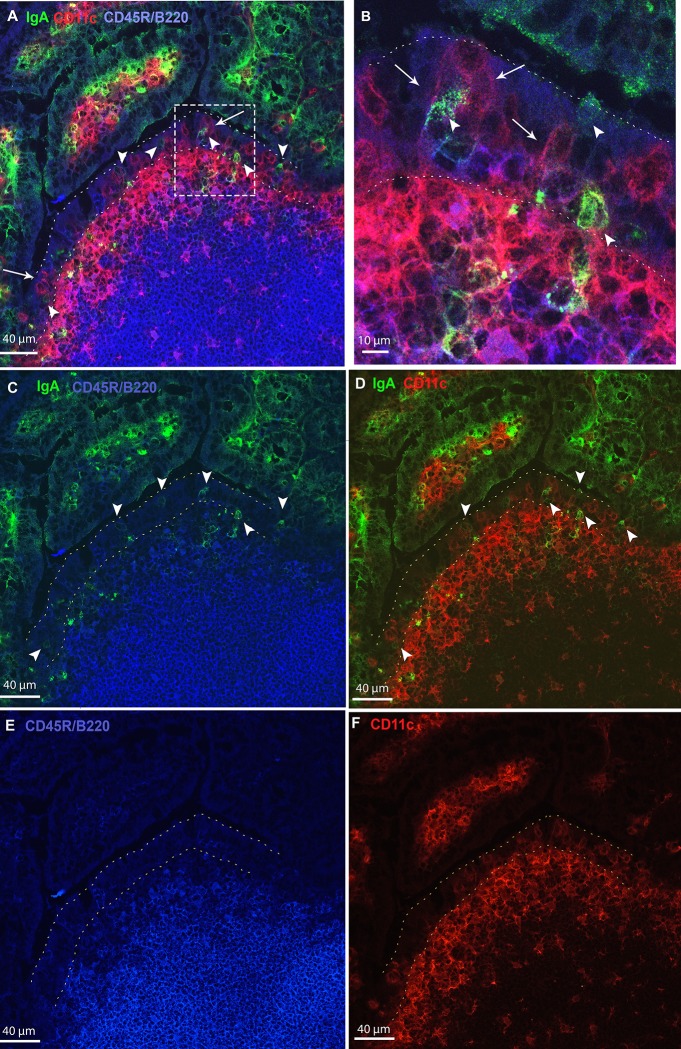
Immunofluorescence staining of B cells, DCs and IgA+ cells in mouse Peyer’s patches. Cryosections of Peyer’s patches from BALB/c mice were stained with antibodies directed against IgA (green,), CD11c (red), and in Panels A and B CD45R/B220 (blue) and in Panels C and D CD11b (blue). Stained cryosections were visualized by CLSM. Panels B and D are magnifications of the dashed boxes in Panels A and C. Panels E and F are single color representations of panels C and D showing CD45R/B220 (blue) and CD11c (red) staining, respectively. DCs are distributed along the FAE (arrows) and in some cases are associated with IgA^+^ cells present in the FAE (arrowheads). B cells expressing CD45R/B220 are frequently seen along the follicle and occasionally in the FAE. CD45R/B220^+^ B cells were not associated with IgA^+^ staining in the FAE. However, B cells, positive for CD11c were closely associated with DCs in the SED region (Panels B, C arrows).

**Fig 4:**
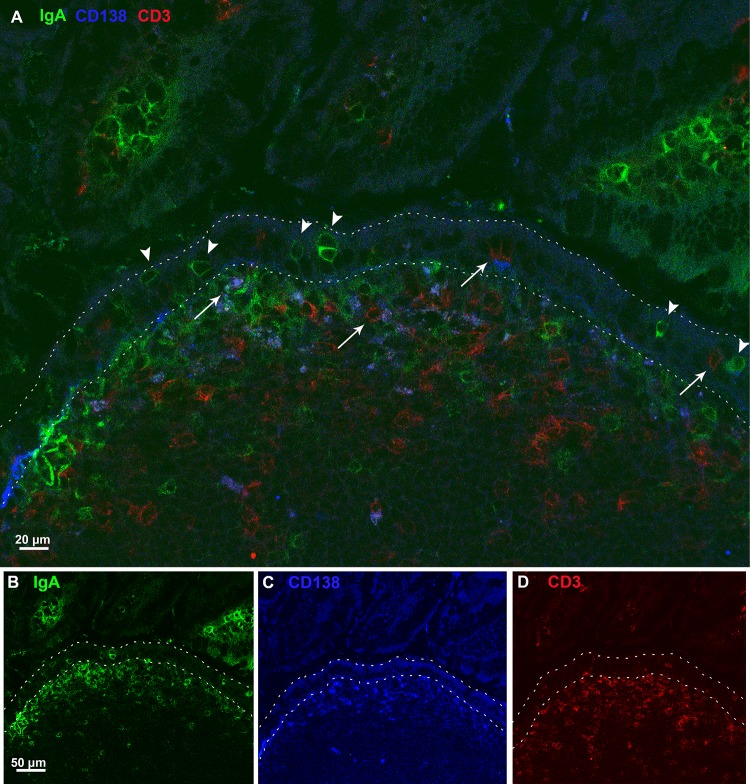
Immunofluorescence staining of plasma cells, T cells and IgA+ cells in murine Peyer’s patches. Cryosections of Peyer’s patches from BALB/c mice were stained with antibodies directed against IgA (green; Panels A, B), CD3 (red; Panels A, C), and CD138 (blue; Panels A, D), and then visualized by CLSM. CD138^+^ cells were mostly found in the SED region where they were commonly associated with T cells and in some cases with IgA^+^ (arrows; Panel A). Along the FAE, CD138 positive staining was rarely observed, and when present it was found in close association with T cells (arrow in FAE region). IgA^+^ cells distributed in the FAE did not colocalize with any of the other two markers used (arrowheads). Panels B, C, D show individual stainings for each marker used in panel A.

In an effort to phenotype the IgA^+^ cells in the FAE and SED, we performed immunostaining of BALB/c Peyer’s patches with a collection of antibodies directed against surface antigen markers for B-cells (CD45R/B220), T cells (CD3), DCs (CD11c), antibody-secreting plasma cells (CD138), and macrophages (CD11b) (Tables 1 and 2). Representative images of an immunostained Peyer’s patches sections are shown in Figs 3 and 4. As expected, B-cells were present throughout the follicle while DCs were most evident within the SED region. Our immunostaining data revealed that the IgA^+^ cells in question did not express the surface antigens associated with B cells, T cells or DCs (see Table 2). CD11b+ staining was readily observed in the lamina propria, but difficult to discern in the FAE and SED, which means the relationship between this specific marker and IgA+ cells remains inconclusive. IgA^+^ cells were, however, often in close association with CD11c^+^ cells, particularly in the FAE (Fig 3B, arrowheads). It is interesting to note that CD11c+ staining was dimmer on cells in the FAE as compared to the SED, a fact that we have observed previously [4]. It is unknown whether this apparent difference in CD11c+ staining is physiologically relevant or an artifact of the immunostaining protocol.

**Table 2:** Summary of antibody labeling in mouse GALT.

		FAE		SED		
Ab/Lectins	LP	IgA+	Total	IgA+	Total	IFR
CD45R/B220 (B cells)	-	-	+	+	++	+++
CD19 (B cells)	+	-	-	-	++	+
CD11b (monocytes and macrophages)	++	-	-	-	+/-	+
CD11c (DCs)	++	-	+	-	+++	++
Ki-67 (Plasmablasts)	+	-	+	++	++	+
CD138 (plasma cells)	+++	-	+	++	++	+
IRF4 (mature plasma cells)	-	-	-	+	++	++
CD3 (T Cells)	+	-	-	-	++	+++
CCR9 (CD199)	-	-	+++	-	+	-
CCR6 (CD196)	-	-	++	-	+++	-
CCL20 (MIP-3α)	++	-	+++	-	++	+
IgG	+++	+	+++	-	+++	+++
IgM	+	-	++	-	+++	++
UEA-1 (M cells)	-	-	+++	-	-	-

Staining key for markers used:

+++ strong

++ frequent

+ occasional

- none

* Indicates only the IgA^+^ subpopulation of cells within the FAE and SED.

We also observed that IgA^+^ cells in the FAE were negative for CD138, while a small fraction of IgA^+^ cells in the SED were positive for CD138, suggesting that a minority of IgA^+^ cells in the SED (but not FAE) are plasma cells (Fig 4). Finally, the IgA^+^ cells in the FAE did not express IFN regulatory factor 4 (IRF4), which is required for the development of early germinal center B cells and antibody secreting plasma cells (S4 Fig) [36]. Finally, we demonstrated that the IgA^+^ cells failed to stain positive for Ki-67 (S4 Fig), indicating that they are not actively proliferating [7].

### Summary and conclusions

The FAE remains an immunological frontier in that the exact subsets of leukocytes that populate this niche and their specific functions in antigen sampling and in regulating primary and secondary mucosal immune responses remain largely unknown. Indeed, exploring the FAE is inherently challenging because within the context of the entire intestinal tract the FAE constitutes only a tiny fraction of the total epithelial surface. *In vitro* epithelial-B cell co-culture models of the FAE have been successful in the reconstitution of M-like cells capable of mediating trans-epithelial transport of viruses and bacteria [37, 38]. However, the co-culture models do not mimic the complex cellularity of the FAE or the multitude of interactions that must exist between the FAE and the cells that reside within the SED. Laser capture microdissection (LCM) has also been employed as a means to profile the FAE [39], but this approach does not reveal cell-cell interactions or cell dynamics that occur within the FAE.

Therefore, in this paper we employed a largely descriptive approach to defining a unique cell subset within the FAE. Using multicolor immunofluorescence confocal microscopy of mouse Peyer’s patch cryosections, we describe a population of IgA^+^ cells that reside within the FAE, as well as in the underlying SED. The IgA^+^ cells were frequently observed to be in close association with M cell basolateral pockets (in accordance with what Farstad and colleagues observed in human Peyer’s patch tissue sections [7]) and with CD11c^+^ DCs, thus making them ideally situated to encounter mucosal antigens and/or antigen-antibody complexes. Moreover, we postulate that the IgA^+^ cells in the FAE are actively migrating into and out of the FAE, based on the observation that IgA^+^ cells were frequently observed on both sides of the basal lamina. Furthermore, in separate studies we have observed that the number of IgA^+^ cells in the FAE increased following exposure of the intestinal mucosa to cholera toxin (M. Hernadez and N. Mantis, unpublished results).

The IgA^+^ cells in the FAE were negative CD138, indicating that they are not plasma cells, although CD11b staining was inconclusive [40]. Based on these observations, we propose that the IgA^+^ cells situated in the FAE are possibly a unique subset of memory B cells involved in antigen surveillance. Very recent advances have defined at least five B cell memory cell subsets in mice based on differential expression of CD80, PD-L2, and CD73 [41–43]. In addition to phenotyping these cells in more detail, in future studies we will take advantage of transgenic B cell receptor mice to begin to examine homing and proliferation of the IgA+ cells in FAE in response to antigen-specific stimulation.

## Supporting Information

S1 Fig Immunofluorescence staining of IgM in murine Peyer’s patches. Cryosections of BALB/c Peyer’s patches were stained with antibodies directed against IgA (green) and IgM (red). The merged image is shown in panel A and the single color files shown to the right. IgM is distributed along the FAE (arrows panel A) and frequently in the SED. IgM is not associated with IgA^+^ cells present in FAE (arrowheads panel A).
(TIF)

S2 Fig Immunofluorescence staining of IgG in murine Peyer’s patches. Cryosections of BALB/c Peyer’s patches were stained with antibodies directed against IgG (blue), E-cadherin (green), and IgA (red,), and then visualized by CLSM. Individual single color stainings are shown in the panels to the right. E-cadherin staining delineates the intestinal epithelium. The FAE is defined with dashed lines. IgA^+^ cells were observed in the FAE (arrowheads), the SED (arrows) and LP (arrows). IgG^+^ cells were observed in the SED and LP but not the FAE.
(TIF)

S3 Fig IgA^+^ cells are present in the FAE of murine Peyer’s patches of BALB/c and pIgR knock-out mice. Cryosections of BALB/c (panels A, C) or pIgR^-/-^ C57BL/6 (panels B, D) mice were stained with antibodies directed against IgA (green) and CD138 (blue) and then visualized by CLSM. Panels C and D are magnifications of the dashed boxes in Panels A and B. IgA^+^ cells are present in the FAE (arrowheads). The apparent CD138 positive staining of the villus epithelium and FAE is likely due to residual background staining and is not considered specific.
(TIF)

S4 Fig Immunofluorescence staining of Ki-67, CCR6, IRF4 and IgA+ cells in murine Peyer’s patches. Cryosections of BALB/c Peyer’s patches were stained with antibodies directed against IgA (red: panels A,B,C; green, panel D), Ki-67 (blue, panels A and B), CD196 for CCR6 (blue, panel C), and IRF4 (red, panel D) and then visualized by CLSM. Ki-67^+^ cells were mostly found in the SED region where they colocalized with IgA^+^ cells (arrows, Panel A). IgA^+^ cells distributed in the FAE did not colocalize with Ki-67 indicating that the IgA^+^ cells in FAE are not actively proliferating (arrowheads, panel B). CD196, marker used to stain for CCR6, was only present in the SED (arrows, panel C) and did not colocalize with IgA^+^ cells in the FAE (arrowheads, panel C). Interferon regulatory factor 4 (IRF4), with roles in mature plasma cell differentiation, was only found through out the SED (arrows, panel D) and was not associated with IgA^+^ cells. IgA^+^ cells in FAE did not colocalize with any of the other two markers used (arrowheads).
(TIF)
