# Supplementary figures and images for: Phenotypic Analysis of a Population of IgA+ Cells in the Follicle-Associated Epithelium of Mouse Peyer's Patches
Source: PLoS One. 2015 Apr 20;10(4):e0124111. doi: 10.1371/journal.pone.0124111 (PMC4404297; doi:10.1371/journal.pone.0124111)

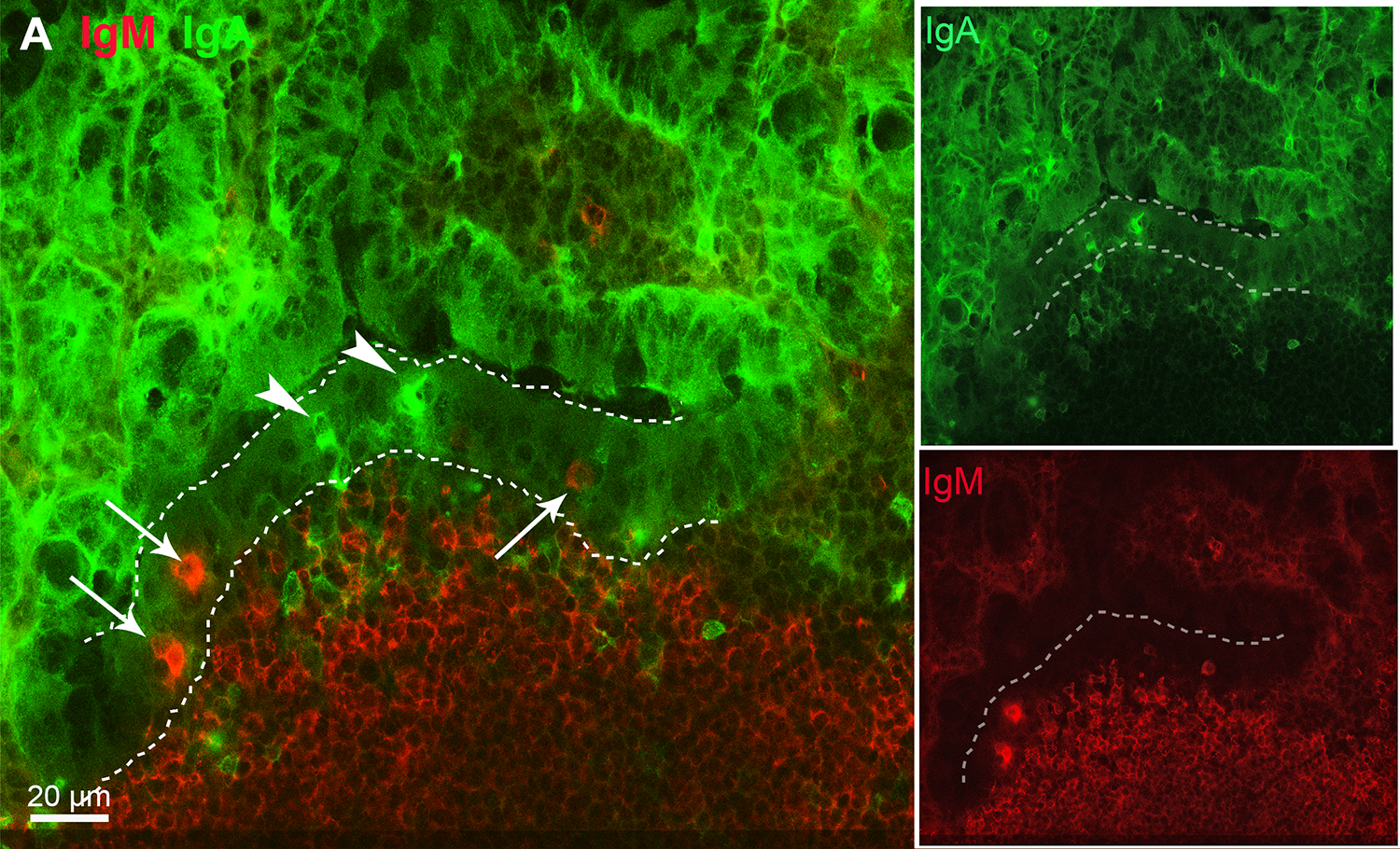

Supplement: S1 Fig — Cryosections of BALB/c Peyer’s patches were stained with antibodies directed against IgA (green) and IgM (red). The merged image is shown in panel A and the single color files shown to the right. IgM is distributed along the FAE (arrows panel A) and frequently in the SED. IgM is not associated with IgA+ cells present in FAE (arrowheads panel A). (TIF) [file pone.0124111.s001.tif]

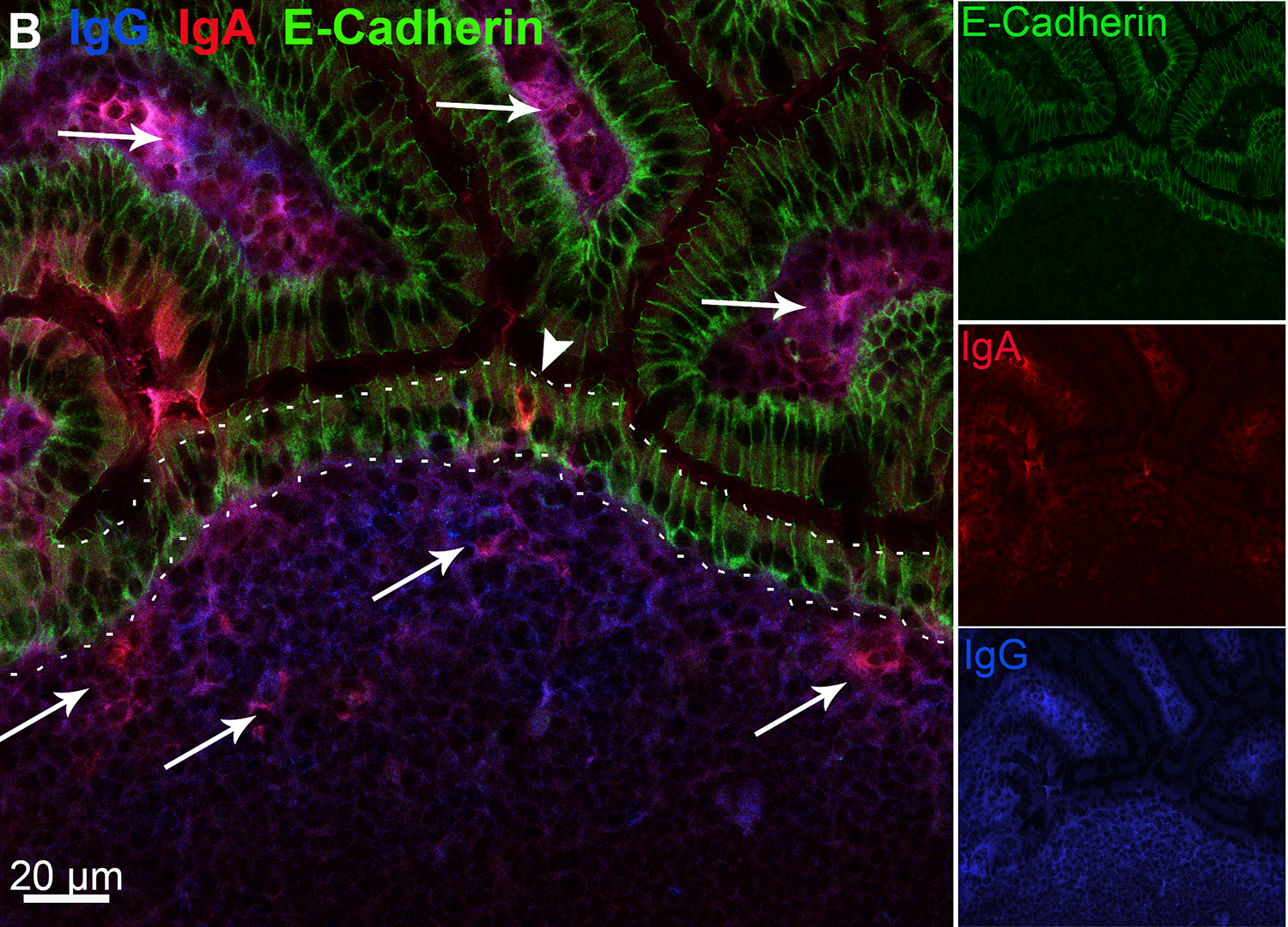

Supplement: S2 Fig — Cryosections of BALB/c Peyer’s patches were stained with antibodies directed against IgG (blue), E-cadherin (green), and IgA (red,), and then visualized by CLSM. Individual single color stainings are shown in the panels to the right. E-cadherin staining delineates the intestinal epithelium. The FAE is defined with dashed lines. IgA+ cells were observed in the FAE (arrowheads), the SED (arrows) and LP (arrows). IgG+ cells were observed in the SED and LP but not the FAE. (TIF) [file pone.0124111.s002.tif]

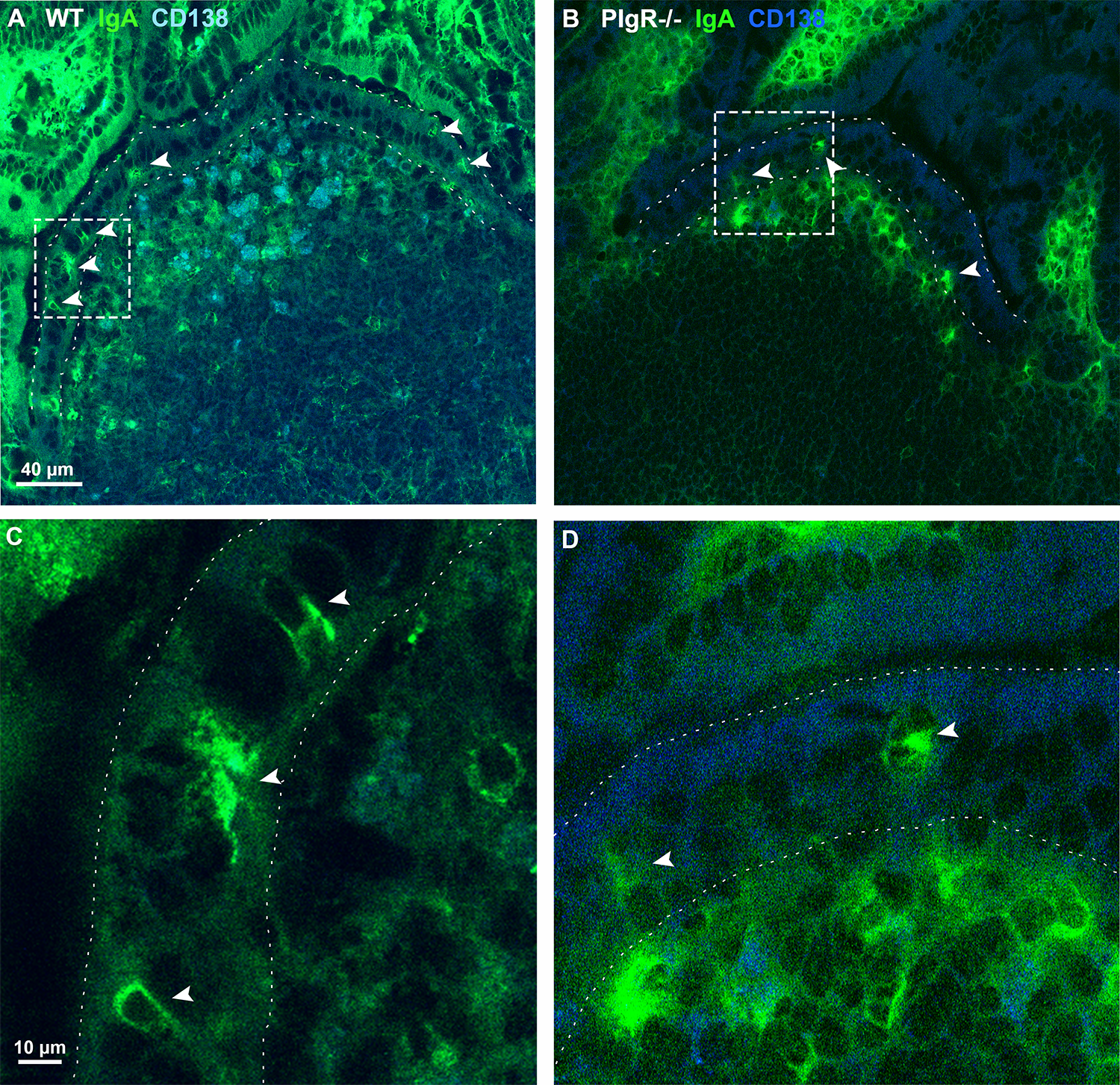

Supplement: S3 Fig — Cryosections of BALB/c (panels A, C) or pIgR-/- C57BL/6 (panels B, D) mice were stained with antibodies directed against IgA (green) and CD138 (blue) and then visualized by CLSM. Panels C and D are magnifications of the dashed boxes in Panels A and B. IgA+ cells are present in the FAE (arrowheads). The apparent CD138 positive staining of the villus epithelium and FAE is likely due to residual background staining and is not considered specific. (TIF) [file pone.0124111.s003.tif]

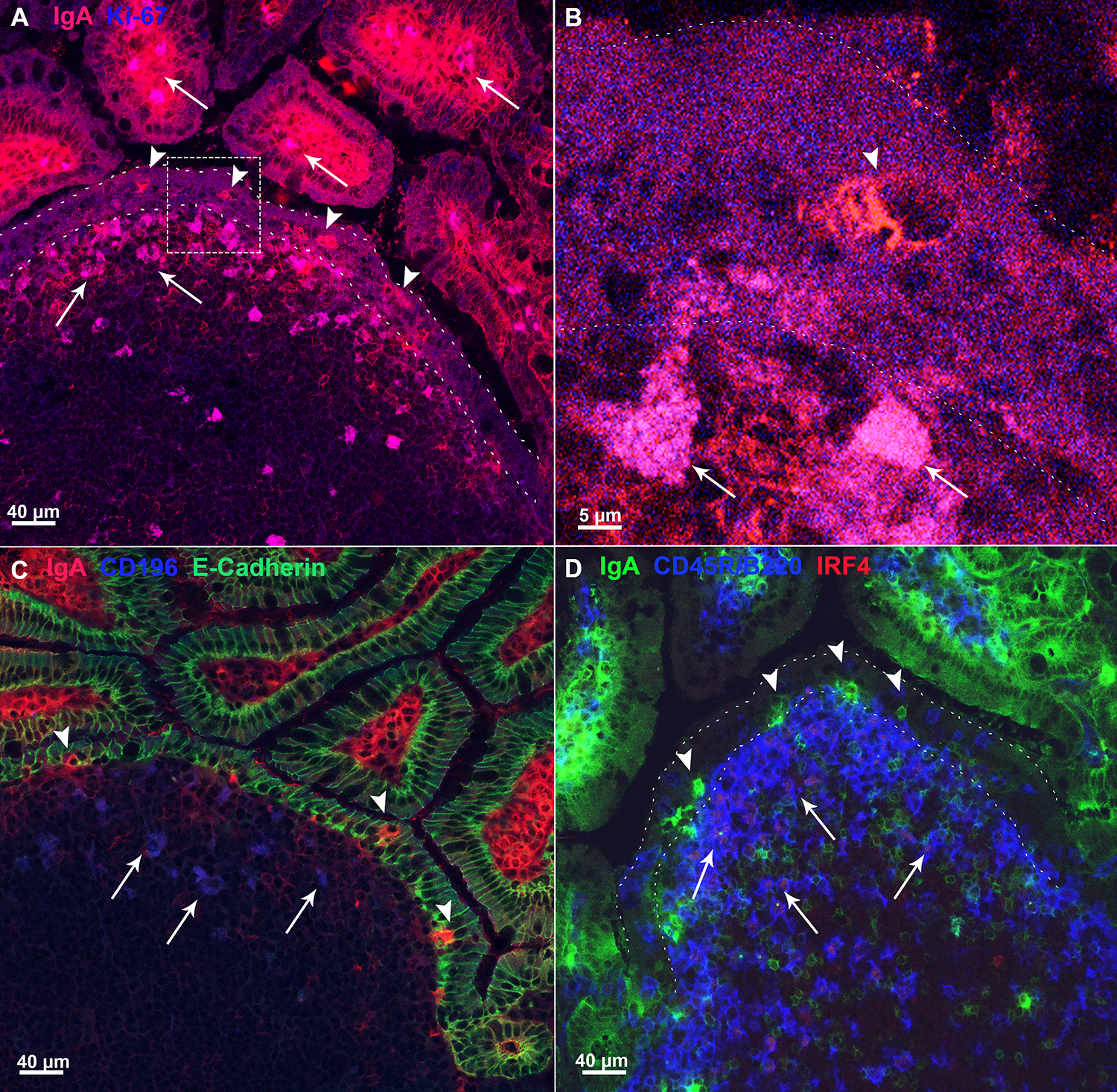

Supplement: S4 Fig — Cryosections of BALB/c Peyer’s patches were stained with antibodies directed against IgA (red: panels A,B,C; green, panel D), Ki-67 (blue, panels A and B), CD196 for CCR6 (blue, panel C), and IRF4 (red, panel D) and then visualized by CLSM. Ki-67+ cells were mostly found in the SED region where they colocalized with IgA+ cells (arrows, Panel A). IgA+ cells distributed in the FAE did not colocalize with Ki-67 indicating that the IgA+ cells in FAE are not actively proliferating (arrowheads, panel B). CD196, marker used to stain for CCR6, was only present in the SED (arrows, panel C) and did not colocalize with IgA+ cells in the FAE (arrowheads, panel C). Interferon regulatory factor 4 (IRF4), with roles in mature plasma cell differentiation, was only found through out the SED (arrows, panel D) and was not associated with IgA+ cells. IgA+ cells in FAE did not colocalize with any of the other two markers used (arrowheads). (TIF) [file pone.0124111.s004.tif]
